# Physicochemical aspects of the tumour microenvironment as drivers of vasculogenic mimicry

**DOI:** 10.1007/s10555-022-10067-x

**Published:** 2022-10-13

**Authors:** Elena Andreucci, Silvia Peppicelli, Jessica Ruzzolini, Francesca Bianchini, Lido Calorini

**Affiliations:** Department of Experimental and Clinical Biomedical Sciences “Mario Serio”, University of Florence, Florence, Italy

**Keywords:** Vasculogenic mimicry, Tumour microenvironment, Hypoxia, Extracellular acidosis, Tumour progression

## Abstract

Tumour vascularisation is vital for cancer sustainment representing not only the main source of nutrients and oxygen supply but also an escape route for single or clustered cancer cells that, once detached from the primary mass, enter the blood circulation and disseminate to distant organs. Among the mechanisms identified to contribute to tumour vascularisation, vasculogenic mimicry (VM) is gaining increasing interest in the scientific community representing an intriguing target for cancer treatment. VM indeed associates with highly aggressive tumour phenotypes and strongly impairs patient outcomes. Differently from vessels of healthy tissues, tumour vasculature is extremely heterogeneous and tortuous, impeding efficient chemotherapy delivery, and at the meantime hyperpermeable and thus extremely accessible to metastasising cancer cells. Moreover, tumour vessel disorganisation creates a self-reinforcing vicious circle fuelling cancer malignancy and progression. Because of the inefficient oxygen delivery and metabolic waste removal from tumour vessels, many cells within the tumour mass indeed experience hypoxia and acidosis, now considered hallmarks of cancer. Being strong inducers of vascularisation, therapy resistance, inflammation and metastasis, hypoxia and acidosis create a permissive microenvironment for cancer progression and dissemination. Along with these considerations, we decided to focus our attention on the relationship between hypoxia/acidosis and VM. Indeed, besides tumour angiogenesis, VM is strongly influenced by both hypoxia and acidosis, which could potentiate each other and fuel this vicious circle. Thus, targeting hypoxia and acidosis may represent a potential target to treat VM to impair tumour perfusion and cancer cell sustainment.

## Introduction

In 1889, Stephen Paget reported the “*seed and soil theory*”: “*When a plant goes to seed, its seeds are carried in all directions; but they can only live and grow if they fall on congenial soil*” [1]. Many years after Paget’s observation, and after important revisions, it became clear that cancer cells get through their progression towards malignancy not only due to the expression of relevant ‘driver’ genes but also as a consequence of epigenetic, transcriptional and posttranslational modifications elicited by the multiple interactions, which cancer cells establish in the so-called tumour microenvironment (TME). TME contains cancer cells and host cells co-evolving dynamically through indirect and direct interactions. Indeed, cancer cells adapt themselves to the various conditions offered by TME and TME is modified according to the secreted products generated by cancer cells themselves. This is a real evolving, well-organised tissue with only one critical project: to assist the survival and proliferation of cancer cells. Indeed, the progression of cancer cells occurs in tissue and is not the result of changing of one individual cell; for this reason, TME represents an essential part of cancer. Thus, we need to consider TME and cancer cells as a single functional entity for elaborating new measures of prevention and therapy [2]. In 2011, Hanahan and Weinberg found it necessary to extend the initial hallmarks of cancer to new two characteristics identified as the genomic instability of cancer cells and inflammation of TME [3]. TME may exert multiple epigenetic effects on cancer cells, and this may happen following the several interactions among cancer cells and host inflammatory and immune cells or host-cell derived factors secreted into TME. These changes in cancer cell phenotype may be transient or associated with a more stable character. Along with this reprogramming, cancer cells under the TME influence might gain the ability to disseminate in distant organs and/or lymph nodes. The multifaceted cascade of metastatic spread of cancer cells consists of several steps including primary tumour cell expansion and invasion into the surrounding host tissues, the angiogenic drift that promotes intravasation into the blood vessels, the survival in the circulation and adhesion on the endothelial cells of a distant organ, extravasation and the final colonisation in the target site. In each step of the metastatic cascade, cancer cells need to adapt their abilities, such as detaching/re-attaching, invading using a mesenchymal or amoeboid style of migration, and adapting their metabolic attitude to survive in a quiescent state or proliferate. All these adaptive characteristics are influenced by host cells, such as endothelial cells, cancer-associated fibroblasts (CAF), immune cells including the various types of tumour-infiltrating lymphocytes, and tumour-associated macrophages (TAM). However, to deeply understand the complex interactions between TME and cancer cells, not only in primary tumours but also in metastatic sites, we also need to consider the local physicochemical gradient of O_2_ tension (pO_2_) and pH. Solid tumours are indeed usually hypoxic and acidic, and such physiochemical aspects of the TME are even sustained by the chaotic tumour perfusion. Tumour vasculature consists indeed of irregular and tortuous branching with a consequent establishment of a heterogeneous blood flow favouring a gradient of hypoxia and acidosis. This microenvironment resembles the characteristic of the stem cell niche.

In such hypoxic and acidic TME, cancer cells change their phenotype accordingly to survive and disseminate. They adapt themselves to the reduced pO_2_ modifying their metabolism but also a series of biological properties which have a profound impact on cancer progression. Further, the Warburg effect (i.e. the aerobic glycolytic metabolism of proliferating tumour subpopulations), as well as the anaerobic metabolism acquired by hypoxic cancer cells, together with CO_2_ hydration by the carbonic anhydrase IX promote the acidification of extracellular pH of solid tumours [4]. The dramatic reduction of pH is also sustained by the reduced lymphatic vessels of tumours [5]. Thus, in solid tumours, we observe a reverse gradient of pH, characterising, in contrast to normal tissue, by a higher intracellular pH and a much lower extracellular pH, ranging from 6.5 to 7.1 [6]. As for hypoxia, changes in intracellular as extracellular pH may influence the behaviour of cancer cells contributing to heterogeneity and plasticity re-adaptation of cancer cells towards malignant progression.

Due to the metabolic reprogramming of cancer cells along the gradient of pO_2_, in several regions of solid tumours, different levels of hypoxia and acidosis come together, even though in some circumstances acidosis was detected in well-oxygenated areas as well. In fact, at a determinate level, hypoxia and acidosis have the ability to induce in cancer cells similar programs, such as genomic instability [7, 8], increased resistance to apoptosis [9, 10] and an epithelial-to-mesenchymal transition (EMT) [11, 12] as stem-like features [13, 14]. In comparison, the metabolic adaptation of cancer cells to hypoxia and acidosis is different, as hypoxic cancer cells undergo anaerobic glycolysis [15] while acidic cancer cells prefer a more respiratory trait, using substrates such as glutamine and free fatty acids [16].

What is particularly intriguing is the role of hypoxia and acidosis in tumour vascularisation. Tumour vasculature can arise from different mechanisms: (1) angiogenesis, consisting of the formation by endothelial cells (ECs) of new blood vessels from existing tissue-resident ones; (2) vasculogenesis, which instead involves EC precursors; (3) intussusception, defined as the vessels splitting through the insertion of tissue pillars; (4) vessel co-option, consisting of the tumour cell migration along the existing vasculature; (5) cancer stem cell (CSC) trans-differentiation to ECs, which in turn generate blood vessels; and (6) vasculogenic mimicry (VM), the phenomenon by which tumour cells mimic ECs and form vascular channels themselves [17–19]. Among them, VM is gaining increasing interest in the scientific community. Being a capacity of the tumour to provide by itself sufficient blood supply for its sustainment [20], VM refers to the ability of aggressive cancer cells to produce fluid-conducting vessel-like structures in an EC-independent way [21]. First discovered in uveal melanoma in 1999 [20], during the following 20 years, VM has been reported in several malignant tumours, including melanoma [22, 23], glioblastoma [24, 25], osteosarcoma [26, 27], hepatocellular carcinoma [28, 29], and breast [30, 31], lung [32, 33], gastric [19, 34], colorectal [35, 36] and prostate [37, 38] cancers. VM is associated with a high tumour grade, invasion, metastasis and poor prognosis in patients with malignant tumours [39–43], including breast [44], colorectal [36], prostate [45], hepatocellular [46], lung [45], ovarian [47], gastric [48] and bladder [49] cancers. To date, the precise mechanisms underlying VM formation are still under investigation; what is known is its correlation with ECM remodelling, certain tumour environmental conditions, and a dedifferentiated state proper of stem-like cells or cancer stem cells (CSCs). Indeed, the ability of VM tumour cells to alter their markers and behave like ECs allows considering them as aggressive tumour cells with high plasticity [50]. Thus, although VM and CSCs-to-ECs trans-differentiation have been proposed as separate ways to form tumour vessels, they could be seen as intertwined pathways towards the same goal. Considering the impact of hypoxia and acidosis on tumour perfusion, we know that hypoxia promotes tumour angiogenesis via HIF-1α signalling, while acidosis induces a reversible mechanism of EC-driven angiogenesis inhibition [51]. However, more importantly, both hypoxia and acidosis stimulate in cancer cells a VM programme [20]. To date, the relationship between VM and angiogenesis remains unclear; indeed, it was found that antiangiogenic therapy does not modify VM [52–54] and VM may sustain tumour growth also during anti-angiogenic therapy [55–57].

Overall, we believe that a reviewing work on the role of hypoxia/acidosis on VM is crucial to outline the dynamic structures of TME useful to establish new adjuvant therapy for tumours.

## Hypoxia and acidosis in TME

Before reviewing the role of acidity and hypoxia on VM, it is useful to summarise the effects of these two biochemical aspects of TME on tumour initiation, development, invasion, metastasis and therapeutic response. Gillies and Gatenby suggest that cell adaptation to this acidic/hypoxic TME represents a key step in the transition from a benign tumour to a malignant carcinoma [58, 59]. The multi-step process of metastatic cascade encompasses local infiltration of cancer cells into the neighbouring tissues, intravasation, survival in the circulatory system, extravasation and subsequent colonisation in distant sites from the primary tumour. Neovascularisation is a fundamental step for tumour development, invasion and spread. Indeed, tumour growth requires the contemporary growth and new formation of a supporting vasculature network to maintain oxygen and nutrient supply to the tumour; moreover, invasive cells need to reside near blood or lymphatic vessels to access the blood circulation or the lymphatic system [60]. Hypoxia, through the induction of HIF-1α, is involved in all steps of blood vessel formation [61]: it contributes to the recruitment of endothelial progenitor cells from the bone marrow and their differentiation into endothelial cells (ECs) by the regulation of the vascular endothelial growth factor (VEGF) and the stimulation of other pro-angiogenic agents such as the VEGF-R2, the members of the FGF family and the platelet-derived growth factor (PDGF) [61]. HIF-1α is also involved in the angiogenic process by the induction of matrix metalloproteinase (MMP) release favouring EC activation, sprouting from pre-existing vessels and proliferation towards a hypoxic area [62, 63]. Finally, hypoxia and HIF-1-α support the creation of mature and stable blood vessels by inducing Ang-1, PDGF and TGF-β to recruit supporting cells such as pericytes and smooth muscle cells [64]. Recent evidence indicates that the HIF-1α-mediated hypoxia response pathway plays a crucial role also in lymphangiogenesis, upregulating the VEGFR-3 ligands VEGF-C and VEGF-D [65].

While the effect of hypoxia on tumour angiogenesis is well known, the interplay between acidosis and angiogenesis is controversial and the effect of low pH on pro-angiogenic factors, such as VEGF-A expression, remains unclear. Shi et al. reported that the incubation of tumour cells at different pH for different times reveals a correlation between low pH and stimulation of VEGF levels [66], while, on the contrary, Scott et al. observed an inverse regulation between acidity and VEGF mRNA expression in breast cancer cells [67]. We contributed to the understanding of the role of extracellular acidosis on lymphangiogenesis, demonstrating that exposure of melanoma cells to low pH (6.7 ± 0.1) promoted the NF-kB-mediated induction of lymphangiogenic growth factor C (VEGF-C) [68], known to be expressed by several aggressive human melanoma cell lines *in vitro* [69]. These data suggest that an acidic microenvironment can influence lymph node metastasis probably by inducing functional changes in lymphatic endothelial cells. In line with this hypothesis, Nakanishi et al. [70] found that extracellular acidosis induces the expression of lymphangiogenic factors, especially IL-8, together with the proliferation of lymphatic endothelial cells. Concerning the effects of extracellular acidosis directly on endothelial cells, we have recently demonstrated that acidosis per se and lactic acidosis are sufficient conditions to dramatically impair *in vitro* EC capillary network/lumen formation and invasion without altering cell viability [51]. In line with our observations, Faes and colleagues observed a reduced EC proliferation and migration in response to VEGF upon acidic exposure [71]. The EC functions’ impairment that we observed under extracellular acidosis was, however, restored following the re-establishment of standard pH conditions, in accordance with Mena and colleagues showing a potentiated angiogenic activity of human endothelial colony-forming cells that have undergone acidic preconditioning and then were re-exposed to standard pH [72, 73].

After the formation of new vessels, tumour cells will get in contact with venules, capillaries or lymphatics and can enter the circulation. To intravasate, tumour cells slow down proliferation, activate anti-apoptotic mechanisms, alter cellular phenotype from epithelial to mesenchymal, reduce cell–cell adhesion and degrade extracellular matrix components, all changes that tumour cells undergo during the EMT process [74, 75]. Several studies have shown the effect of hypoxia and acidic pH in inducing EMT in tumour cells, selecting cells with high plasticity, enhanced ability to invade [11, 12] and greater survival, by suppressing apoptosis [9, 10], promoting autophagy [76, 77] and avoiding anoikis cell death when cells are detached from extracellular matrix [78, 79].

There is a strong linkage between hypoxia, acidity and stemness. Low O_2_ concentration has been closely associated with the maintenance of a stemness trait in tumour cells residing in specific stem cell niches [13]. Several studies have demonstrated that acute hypoxia selects for cancer cells with stem cell characteristics, enhancing stem-like cell marker expression, such as OCT4, SOX2, NANOG and MYC, through upregulation of the HIF-1α pathway [80, 81]. Moreover, increased expression of HIF-1α and HIF-2α has been found in stem cell–like populations of neuroblastomas [82] and gliomas [83]. Recently, we contributed to the understanding of the role of an acidic TME in cancer cell stemness demonstrating that exposure to low extracellular pH induces the expression of pluripotency markers such as Nanog, KLF4, OCT4 and Sox2 and the over-expression of stemness markers such as CD133, CD243 and ALDH1A1 in melanoma cells [14]. The correlation between acidity and stemness was also verified in prostate cancer [84], malignant glioma [85, 86] and osteosarcoma [87], and it is sustained by NF-kB activation [14, 87, 88].

Tumour hypoxia and extracellular acidosis exert on cancer cells a selective pressure able to lead to the propagation of subpopulations characterised not only by an aggressive phenotype, but also by the ability to resist apoptotic stimuli, for example, after pharmacological treatment. In 1953, Gray et al. [89] demonstrated that hypoxia can confer resistance to radiotherapy. Moreover, several studies show the effect of low oxygen in increasing resistance to chemotherapy leading to tumour recurrence and ultimately limiting patients’ prognosis [90–92]. Also, acidity is a contributing factor in the resistance of tumours to therapy. Thews et al. found an enhancement of pGP activity in acidic prostate carcinoma cells caused by an increase of intracellular Ca^2+^ levels and related to ERK and p38 pathway [93]. Several other mechanisms of low pH-induced drug resistance have been proposed; one of these has a chemical nature and it is called ‘ion trapping’, based on the ion protonation of drugs [94]. It has been widely demonstrated that low pH reduces uptake and efficacy of weak base chemotherapeutics such as anthracyclines, anthraquinones and vinca alkaloids [95, 96], thus alkalisation could be useful to restore cell drug sensitivity. Autophagy, a self-digestive process that cells perform in response to nutrient stress, is now considered a mechanism of drug resistance in cancer; indeed, it has been found to maintain cancer stem cell phenotype and correlates with chemo- and radio-resistance [89, 97–100]. It was demonstrated that hypoxia can induce autophagy in different cellular settings [76, 101], acting as a survival mechanism for hypoxic cells through the recycling of cellular components. Wojtkowiak et al. proved that autophagy is induced also in response to acute and chronic acidity as a cellular adaptation to survival and that an autophagy inhibitor, 3-methyladenine, could be used to affect acidic cells [77], suggesting that autophagy could represent a valid therapeutic target. Also, other studies demonstrated that proton pump inhibitors which inhibit extracellular acidification, such as pantoprazole, lansoprazole and omeprazole, or buffering experiments using sodium bicarbonate, can suppress the later stages of autophagy [102, 103].

Both hypoxia and acidity-driven adaptive mechanisms allow tumour cells to continue to survive in the hypoxic/acidic TME while also creating an inhospitable environment for immune cells and damaging key regulatory pathways [104, 105], inducing immune suppression and contributing to a reduced anti-tumour response. Immune surveillance represents the first line of defence against cancer, but often cancer cells manage to escape it. Indeed, upon exposure to acidosis or hypoxia, tumour cells release a large variety of immunosuppressive molecules, such as IL10, TGFβ and VEGF, that act on the immune system in three main ways: inhibiting immune cell proliferation and survival [106, 107], affecting immune cell function [106, 108] and regulating the signalling of downstream processes, such as PDL-1 overexpression [109, 110]. An important reason for immune disfunction in the acidic condition is that the elevated glycolytic activity of tumour cells reduces glucose availability and activated T cells do not survive without glucose, differently from tumour cells that enter quiescency [111]; especially CD8^+^ T cytolytic activity results significantly reduced by acidification [112]. Also, hypoxia was shown to decrease T-cell survival [113] and proliferation [114]; Lukashev et al. [115] demonstrated that CD4^+^ and CD8^+^ T cells derived from HIF-1α-deficient mice show increased proliferation, can secrete higher levels of interferon-γ and exhibit enhanced antitumour responses. Moreover, hypoxia has been shown to promote regulatory T cell (Treg) induction [116]. Acidosis and hypoxia not only decrease T-cell functionality, but they also impair dendritic cell maturation [117, 118], lymphokine-activated killer cells [119, 120] and natural killer (NK) cells [121, 122]. Colegio et al. [123] and Ke et al. [124] found respectively that low pH and hypoxia are responsible for TAM polarisation to M2 and the induction of VEGF production. Similarly, other authors found a correlation between the acidic/hypoxic microenvironment and the release of pro-angiogenic mediators by macrophages [125, 126]. Recent studies demonstrated *in vivo* that neutralisation of tumour acidity using oral buffers [127] or treatment with proton pump inhibitors [128] respectively ameliorates T-cell activity and TAM activation in the tumour microenvironment.

Accumulating evidence indicates that metastasising cells undergo dynamic metabolic changes, a necessary step contributing to their plasticity, useful to successfully survive the microenvironmental changes during the metastatic cascade [129]. Under hypoxic conditions, HIF-1α triggers the expression of genes involved in the metabolic shift towards anaerobic glycolysis, such as pyruvate dehydrogenase kinase 1 (PDK1) to decrease mitochondrial oxygen consumption [130], hexokinase 2 (HK2), lactate dehydrogenase A (LDHA), pyruvate kinase isoform M2 (PKM2) and enolase1 (ENO1), and promotes the expression of glucose transporters (GLUT1 and GLUT3) [15, 131]. In addition, hypoxic cancer cells activate the glycogen synthesis pathway to reserve glucose. Another enzyme involved in glycolysis, PFKFB4 (6-phosphofructo2-kinase/fructose-2,6-biphosphatase 4), was demonstrated to be upregulated following hypoxia exposure, and its high expression correlates with poor prognosis [132]. Thus, the HIF-1α pathway increases glucose uptake and secretion of lactate, which accumulates in hypoxic cells together with H^+^. These products are exported via monocarboxylate transporter 4 (MCT-4), carbonic anhydrase (CA9) and Na^+^/H^+^ exchanger (NHE-1) which are all under HIF-1α control [133]. Hypoxia, through HIF-1α, regulates also glutamine metabolism; it causes reduced glucose-derived citrate by decreasing PDH activity and an increase in aKG levels induced by the reduction of aKGDH activity [134]. In contrast to hypoxia, which induces glucose uptake and metabolism, tumour acidosis leads to a dramatic reduction in the use of glucose by tumour cells [16], shifting the metabolism to oxidative phosphorylation (Oxphos) with the decrease of glycolic markers, such as GLUT1, GLUT3, HK2, G6PD, PKM2 and LDHA, and the increase of CytC and PGC1α We demonstrated that this metabolism can be reverted using metformin, a biguanide commonly used for type 2 diabetes with a mitochondrial antagonist activity [135], and that Oxphos is sustained by the expression of SOX-2 increased by acidic conditioning [136]. The same metabolic behaviour in acidic conditions was found in breast carcinoma cells [16] and malignant neuroblastoma cells [137]. Oxidative metabolism allows cells to get more bioenergetic molecules instead of biomass for cell division [16, 135], and the main energetic source of acidic pH-adapted cancer cells derives from fatty acid oxidation [138]. Corbet et al. [138] demonstrated that under chronic acidosis, acetyl-CoA generation derives from fatty acids and glutamine, instead of glucose. Moreover, several studies have identified HIF2α as a key driver of metabolic adaptation to tumour acidosis [85, 139], while the synthesis and activity of HIF-1α were generally found reduced [139, 140].

## Hypoxia and acidosis in VM promotion

**Fig. 1:**
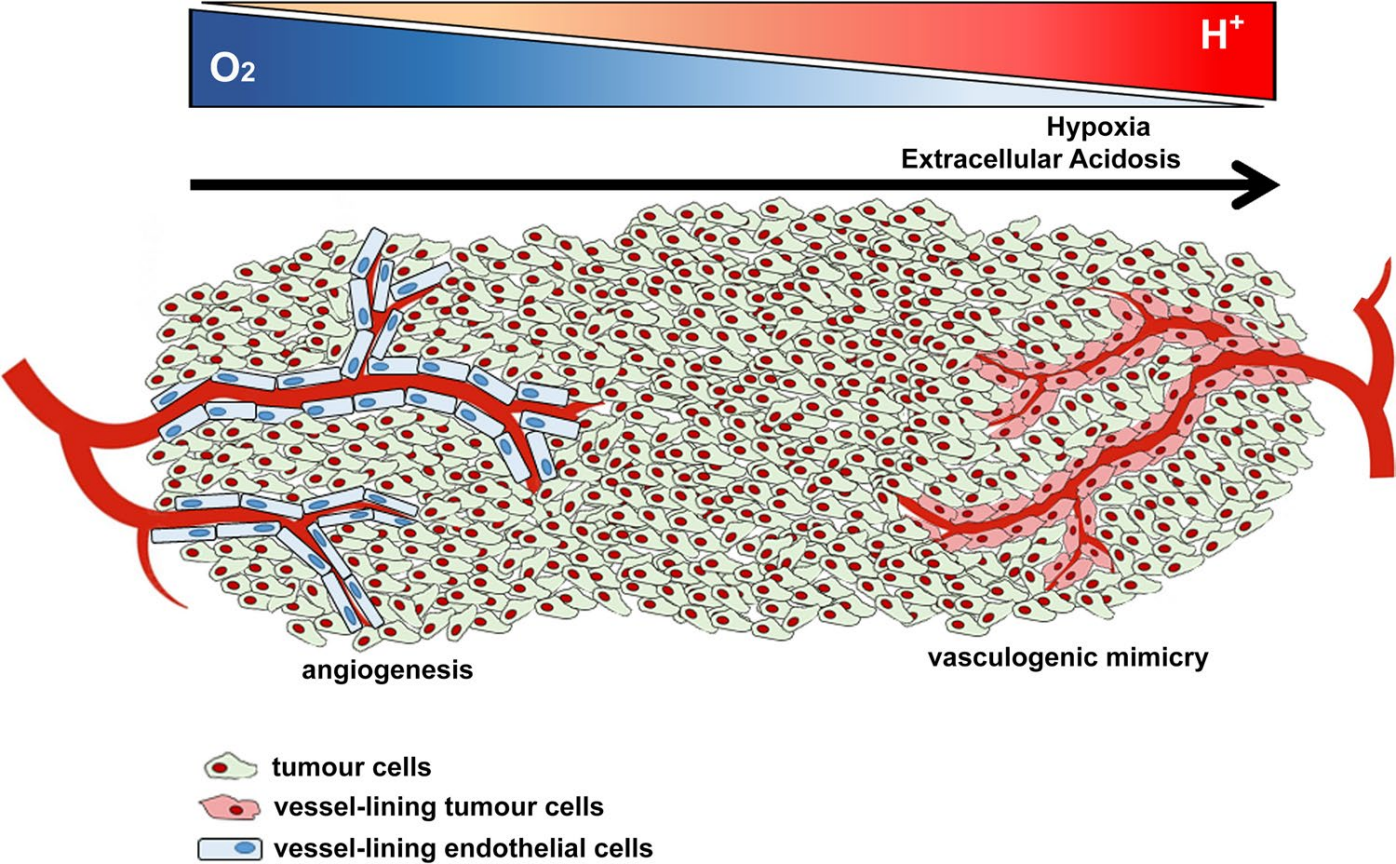
Hypoxia and extracellular acidosis of the tumour microenvironment induce vasculogenic mimicry. As the tumour mass grows, the support of oxygen provided by angiogenic vessels (left) decreases, and, at the meantime, H^+^ accumulate generating a hypoxic and acidic tumour microenvironment. Such a condition induces cancer cell vasculogenic mimicry (right) to support tumour survival and disease progression

VM is finely regulated by hypoxia and acidosis of the TME (Fig. 1). Before going deeper into the mechanisms through which hypoxia and acidosis induce VM, let us briefly analyse the principal actors in this phenomenon.

### VM regulating pathways

Two of the first proteins identified as central players in VM are VE-cadherin, a cell–cell adhesion molecule expressed by ECs allowing adherent junction formation [141], and EphA2, an epithelial cell receptor tyrosine kinase involved in EphA1-induced angiogenesis and found upregulated in a wide variety of cancers, whose expression has been linked to increased malignancy and a poor clinical prognosis [142]. Several studies appositely designed to disclose the contribution of these two proteins in VM allow understanding that they act in a coordinated way since VE-cadherin regulates the EphA2 localisation at the intercellular junctions between VM-forming tumour cells as well as its phosphorylation level [42, 143]. The signalling triggered by the kinase EphA2 leads to FAK-mediated PI3K and ERK1/2 pathway activation, which besides being associated with survival, proliferation and migration are also important in the VM process; in fact, PI3K regulates extracellular matrix remodelling, crucial for VM-forming tumour cell migration, by inducing the activation of MMP-2 (mediated by MMP-14) and MMP-9. On the other hand, ERK1/2 signalling has been found to upregulate urokinase and MMP-2 activity and thus stimulates the invasion of VM-forming tumour cells [42, 144, 145]. VE-cadherin expression is also regulated by Twist-1, at least in hepatocellular carcinoma, astrocytoma and gastric cancer [146–148].

A discussed player of VM is the VEGF-A. Considered the main pro-angiogenic factor—for stimulating EC proliferation, survival and angiogenesis—there is evidence supporting its involvement in VM promotion, at least in melanoma and ovarian cancer [149, 150]. Among the signalling pathways triggered by VEGF-A binding to VEGF-R1, the activation of the tyrosine kinases Src and ERK1/2 leads to the promotion of cancer cell invasion and migration, known to be crucial steps for VM. Also, in melanoma, PI3K activation triggered by VEGF-A/VEGF-R1 binding contributes to VM by stimulating MMP-14-mediated MMP-2 activation and subsequent tumour cell migration and tube formation [42]. In ovarian cancer, VEGF-A/VEGF-R1 signalling increases the expression level of VM-specialised genes such as VE-cadherin, EphA2 and matrix-metalloproteinases MMP-2 and MMP-9 [151]. On the contrary, an opposite point of view has been proposed as well, based on the observation that VM increases in the absence of VEGF-A signalling: VEGF-A blocking strategies increase VM together with other alternative survival ways exploited by aggressive tumour cells [22]. Rather than VEGF-A blockage/absence itself, this could occur because upon VEGF-A inhibition and subsequent VEGF-A-driven angiogenesis impairment, the tumour microenvironment where cancer cells grow likely becomes increasingly hypoxic/acidic, conditions that stimulate the VM-driven tumour vascularisation. Focusing on the VEGF-R1, it has been demonstrated that the signalling it triggers is required for VM of melanoma cells to express VE-cadherin upon VEGF-A stimuli [149]. Even Cyclooxygenase-2 (COX-2) has been proven to induce VM by stimulating the VEGF-A expression [42, 152, 153]. COX-2, the enzyme that converts arachidonic acid into prostaglandin E2 (PGE2), upon binding prostanoid receptors (EP1–4), activates PKC signalling that in turn stimulates VEGF-A expression and subsequent tumour-derived vascular channel formation in glioblastoma, and lung and breast cancer [33]. Furthermore, the COX-2 product PGE2 signalling activates the EGF receptor (EGFR) with PKC-dependent ERK1/2 activation. Consequently to this signalling pathway, tumour cell proliferation, invasion, angiogenesis and VM are promoted [154]. The capacity of COX-2 to stimulate VM is in line with the observation that the inflamed tumour microenvironment and infiltration by the tumour-associated macrophages (TAMs) enhance VM-vessel network formation [155, 156]. Keeping the focus on the VEGF family and VM, in melanoma, it has been observed that endothelin-1 (ET-1) increases the expression levels of VEGF-R3 together with its ligands VEGF-C and -D triggering VM signalling [157].

Going further, the players of VM, Tissue Factor (TF) and TF pathway inhibitors 1 and 2 (TFPI-1/2) have been found to be crucial for tumour cell-lined tube formation [42]. These proteins are finely involved in the coagulation pathway. In particular, the TF represents the cell surface receptor that initiates the coagulative cascade: upon binding the VIIa cofactor, the TF activates factor X, triggering haemostasis through downstream thrombin generation and subsequent fibrin formation and platelet activation. The TF is inhibited by the TFPI-1, which, by simultaneously binding the VIIa and Xa factors, locks TF into an inactive TF-VIIa-Xa-TFPI-1 complex. On the contrary, the TFPI-2, originally identified by TFPI-1 homology as placental trypsin and serine protease inhibitor associated with tumour cell ECM [158–160], does not physiologically exert any inhibitory activity on the TF pathway [161]. TFPI-2 is instead a potent inhibitor of plasmin and MMPs that can regulate the adhesion and migration of endothelial and tumour cells in a context-dependent manner [161]. TF, TFPI-1 and TFPI-2 are overexpressed by tumour cells, and experimental evidence highlights their role in VM. TFPI-1 was observed to serve as the predominant regulator of TF activity in aggressive melanoma cells (behaving thus as endothelial cells), and its anticoagulant activity could account for the fluid-conducting capacity of VM tumour cell-lined vessels. On the other hand, studies conducted with anti-TFPI-2 antibodies or with recombinant TFPI-2 highlight the crucial role of the TFPI-2 in the VM-vessel formation, linked with its complex MMP-2 activating function [161].

The Notch family has been found important in the VM process. It comprises four isoforms of transmembrane receptors (Notch1–4) and five possible membrane-bound ligands, Delta-like 1/3/4 and Jagged 1/2. Notch signalling is crucial for embryonic pluripotency and development. Briefly, the Notch intracellular domain (NICD) is released into the cytoplasm and localised in the nucleus after the Notch receptor is sequentially degraded. The role of Notch signalling in VM has been reported in melanoma, gastric cancer and hepatocellular carcinoma [39, 162–164]. In particular, Notch4 was found to induce VM in melanoma cells in a Nodal-dependent way since the treatment with recombinant human Nodal was able to rescue VM ability impaired by Notch4 inhibition [165].

Also, the Wnt/β-catenin signalling pathway is involved in VM formation. It has been reported that Wnt/β-catenin signalling disruption decreases VM in colon cancer, triple-negative breast cancer, melanoma and osteosarcoma [166–170].

The mammalian target of rapamycin (mTOR) represents a crucial player in VM. As we recently reported, mTOR targeting by everolimus inhibits EphA2 and VE-cadherin expression and strongly impairs the ability of melanoma cells to perform VM [145]. Also, in glioblastoma, mTOR-specific inhibitor rapamycin and mTOR silencing were found able to completely disrupt the VM formation under both hypoxic and normoxic conditions [171].

Another important inducer of VM is the EMT. EMT is characterised by the loss of epithelial traits and the concomitant gain of mesenchymal phenotypes. It is accompanied by the expression of the transcription factors Twist, Snail, ZEB1 and ZEB2 which, upon binding to the E-cadherin promoter, mediate its inhibition in favour of an increased tumour cell invasion ability. Much evidence highlights that EMT plays a crucial role in VM, and EMT-related transcription factors are involved in the VM process. Indeed, as reviewed by Liu and colleagues, along with controlling the E-cadherin/N-cadherin and vimentin levels, Twist, Zeb1, Snail and Slug also modulate VE-cadherin and VEGF-R2 expression, activate MMP-2 and MMP-9, as well as guarantee stemness maintenance in VM‐positive cancer cells [50].

### VM regulation by hypoxia and acidosis of the TME

#### Hypoxia

Strong experimental evidence has demonstrated the major role of hypoxia in VM promotion. Under low oxygen conditions, HIF-α protein is stabilised and translocates into the nucleus where it binds the gene regulatory regions containing the hypoxia response elements (HREs) and subsequently activates the transcription of hypoxia-target genes. Hypoxia is a master regulator of several HIF-α-mediated signalling pathways inducing VM development in solid tumours [17, 42]. As reviewed by Seftor and colleagues, several genes involved in VM are directly (through the HIF-α binding to the HREs) or indirectly (via the involvement of an intermediate regulating protein or post-translational modifications) modulated by hypoxia. Among them, hypoxia directly modulates VEGF-A, VEGF-R1, EphA2, Twist and COX-2, and indirectly, VE-cadherin, TF and Notch [143]. Following the knockdown of HIF-1α, VE-cadherin, EphA2 and laminin-5γ2 are inhibited in oesophageal cancer [172]. Hypoxia can modulate the expression of Notch-responsive genes—such as Nodal—via HIF-1α-mediated NICD stabilisation. This non-canonical crosstalk between HIF-1α and Notch may serve to promote tumour cellular undifferentiation and plasticity characterising VM-forming cells. Hypoxia also promotes VM via reactive oxygen species (ROS) generation. Indeed, in melanoma, VM is induced upon redox-dependent stabilisation of HIF-1α [173] These studies demonstrating the hypoxia-induced VM and VM-associated genes also highlight the critical role exerted by hypoxia in tumour migration since it also activates MMP-2 and MMP-9, thus promoting tumour cell invasion to adjacent tissue [174]. In melanoma, it was observed that hypoxia stimulates the anti-apoptotic protein B-cell lymphoma 2 (Bcl-2) which in turn increases VE-cadherin expression [175]. VE-cadherin expression, whose modulation under hypoxia can be dependent on both the isoforms 1 and 2 of HIF-α (HIF-1α and HIF-2α) [176], is also indirectly increased by hypoxia-induced BNIP3, a protein belonging to Bcl-2 family that contributes to VM by promoting cancer cell migration and cytoskeleton organisation during tube formation [175]. Bcl-2 and Bcl-XL proteins were also found to cooperate with hypoxia to induce MMP-2 expression in melanoma [177]. Moreover, HIF-1α and HIF-2α promoted *in vitro* VM tube formation by upregulating the VEGF- C and -D together with their receptor VEGF-R3 [172]. Hypoxia is also an inducer of the EMT and EMT-related transcription factors further enhancing VM as described above [178]. Upon hypoxic tumour microenvironment and subsequent HIF-1α stabilisation, the urokinase-type plasminogen activator receptor (uPAR) was found stimulated as well [179]. It is especially known for its pro-angiogenic activity, but recent findings highlight its role even in VM [145, 180, 181], reinforcing uPAR’s prognostic value in cancer progression and vascularisation [182].

#### Extracellular acidosis

Despite much evidence in the literature linking hypoxia to VM, very little has been reported to date about the contribution of the acidic tumour microenvironment in tumour-derived vessel network formation. We recently demonstrated that acid-adapted melanoma cells are endowed with a higher ability to organise themselves in a capillary-like network. The increased VM ability that we observed in acid-adapted melanoma cells is driven by uPAR and goes with an aggressive tumour phenotype endowed with drug resistance [145]. Interestingly, by studying the effects of the acidic tumour microenvironment on VM and angiogenesis, we observed that if, on one hand, extracellular acidosis severely impairs EC-derived vessel formation [51], on the other hand, it strongly promotes vascularisation from tumour cells themselves [145]. Considering the fast proliferative rate of cancer cells and their boosted glycolytic metabolism, we could imagine a rapidly growing tumour mass with an acidified extracellular milieu which confers several aggressive features including the ability to organise itself in tumour-derived vessels, accomplishing tumour mass vascularisation even in a context where angiogenesis is inhibited or just slowed down. VM likely represents an alternate/complementary route compared to angiogenesis exploited by aggressive tumours to guarantee perfusion and thus sufficient nutrients and oxygen supply needed for the tumour mass expansion. VM can exist just for the period needed by ECs to perform angiogenesis and substitute VM-derived vessels. Indeed, as reported by Zhang and colleagues, VM seems to provide blood supply in the early stages of tumour expansion, while as the mass grows, tumour cells lining the VM vessel wall are little by little, but completely, replaced by ECs, creating a middle transitional phase where both tumour and ECs line the vessels in the so-called mosaic vessels [41]. Indirect evidence also supports the contribution of extracellular acidosis in VM. Indeed, acidosis in recent years has been defined as a new hallmark of cancer and demonstrated to endow tumour cells with aggressive tumour EMT phenotype characterised by a high plasticity, apoptosis resistance, immune surveillance evasion, increased resistance to radio-, chemo- and target therapies, enhanced migration/invasion and metastatic dissemination. So, VM becomes a part of this complex aggressive and plastic tumour phenotype selected by the acidic microenvironment and drastically able to pursue disease progression. Much evidence also links hypoxia and extracellular acidosis in VM promotion since crucial VM promoting pathways are shared by these environmental conditions: (1) EMT, initially, is indeed promoted by both hypoxic and acidic conditions [183]; (2) uPAR is over-expressed under hypoxia but also in the presence of low extracellular pH [145]; (3) mTOR is up-regulated under acidic conditions [184] and, in turn, mediates HIF-1α expression [42]; (4) last but not least, both the hypoxic and acidic tumour microenvironments are important for selecting the CSCs and maintenance of stemness [14].

## Final scenario and possible new strategies for treatment

The activation of a VM program is a paradigmatic example of how cancers deploy their devastating strategy. The emergence of VM is not only part of the natural history of cancer progression but also occurs wickedly as the consequence of anti-tumoural treatments. Accordingly, particular attention must be devoted to planning treatments that target VM.

As part of the natural history of cancer, VM is considered a marker of malignant progression; indeed, this discontinuous and leaky vessel network is strongly associated with aggressiveness and poor prognosis in different types of cancers. In this context, the discovery of novel VM biomarkers will facilitate the diagnosis and clinical evaluation of cancer patients during their treatments. The identification of novel markers will improve treatment monitoring for anticipating the emergence of drug resistance and, consequently, will help physicians in changing the therapeutic strategy when needed. Alongside classic biomarkers of VM such as the overexpression of VE-cadherin (CD144), the periodic acid-Schiff (PAS) positivity [185, 186] and the cell surface glycoprotein CD133, novel biomarkers have been recently identified belonging to the PI3K/MMPs/Ln-5γ2 signalling pathway, to the EphA2/FAK/Paxillin signalling pathway and to the Sp1-dependent twist/VE-cadherin/AKT signalling pathway [56, 187, 188]. Importantly, the critical step in VM-marker future research should consider the discrepancy that exists between cancer patients and xenograft models. Indeed, VM, although undoubtedly detected in cancer patients, appears not so well developed as that developed and detected in xenograft tumours. Two interrelated factors contributed to this: the boosted tumour expansion in xenografts that still overwhelms murine angiogenesis, and, at the same time, a hypoxic and acidic microenvironment that strongly favours VM in xenografts [189].

In addition to the spontaneous appearance of VM during tumour progression and its role as a marker for histopathology, VM occurs in response to anti-tumoural treatments, sustaining the acquisition of a therapy-resistant phenotype in cancer cells [22, 190]. Among the different anti-tumoural treatments, anti-angiogenic treatment reveals the most dangerous and tricky features of VM. In this context, two different aspects must be mentioned. The first is that anti-angiogenic treatments are reported to be the cause of VM development [52]; the second, conversely, is that anti-angiogenic treatments might be very useful against the development of VM and cancer progression. Recently, methionine aminopeptidase-2 (MetAP2), which is involved in the angiogenic process, but also has a pivotal role in VM, became an interesting dual target. Indeed, the use of angiogenic inhibitors, such as fumagillin and TNP-470, interfering with MetAP2, significantly suppressed VM in various human cancer cell lines [191, 192]. Sunitinib, a potent anti-angiogenic agent, represents another interesting example of how tricky the use of anti-angiogenic treatments is. Sunitinib is used in the treatment of renal cell carcinoma (RCC). In RCC cells, the treatment with sunitinib promotes *in vivo* tumour resistance*,* which is reduced with everolimus in second-line treatment [53]. Interestingly, during sunitinib treatment of RCC, the emergence of VM is used as a known target to be exploited, revealing the increasing potential efficacy of sunitinib [193]. Keeping in line with this approach, additional anti-angiogenic treatments that revealed promising efficacy against VM should be mentioned. Preclinical investigations demonstrated that Arg-Gly-Asp integrin antagonists such as cilengitide inhibited angiogenesis and VM. An interesting lesson should be learned from cilengitide. When used as a single agent, this cyclic pentapeptide has shown minimal clinical efficacy in patients with metastatic melanoma. Conversely, if used in combination therapies, it demonstrated antiproliferative effects against melanoma and endothelial cells, both *in vitro* and *in vivo* [194]. Moreover, RGD functionalised nanoparticles targeting αvβ3 integrin expressing cells efficiently and simultaneously inhibit angiogenesis and VM [195, 196]. In this scenario, RGD-conjugated drugs might exert a versatile anti-angiogenic/anti-VM effect [197–199].

We explored the role of TME in VM emergence, and we have extensively mentioned how extracellular acidity and hypoxia play a crucial role in this process. Accordingly, therapeutic strategy devoted to inhibiting extracellular acidity, hypoxia and/or EMT signalling pathways revealed also their inhibitory effect on VM [200]. The knockdown of ZEB1, one of the major inducers of EMT, or the re-expression of ZEB1-repressed microRNA (miRNA) clusters, inhibits VM [201]. The knockdown of Neuropilin-1, which is up-regulated by a HIF-1α-dependent mechanism, inhibited VM formation [202]. Also, the inhibition of the Wnt/beta-catenin pathway, which correlates with EMT, promotes cancer drug resistance and is strongly associated with VM, is used for VM treatment [203]. In view of this, the development of a selective inhibitor directed towards VM reveals as an extremely challenging mission. Conversely, to fight the emergence of VM, a preclinical investigation should consider a drug re-purposing strategy. Drugs that are already developed and approved might reveal a novel application in this field [204].

Finally, there is a growing interest in the use of phytochemicals and naturally derived compounds for the inhibition of VM, in view of their wide spectrum of pharmacological activity [205]. It is well known that natural compounds affect multiple signalling pathways, revealing to be interesting multi-target drugs in different types of diseases. This is the case of curcumin, derived from the rhizome of *Curcuma longa*, and recognised as a bioactive compound responsible for numerous pharmacological activities, one of which is the suppression of VM in hepatocellular carcinoma [206]; of ginsenoside, extracted from ginseng, with anticancer activity against several types of cancers which inhibit VM through the downregulation of VE-cadherin/EphA2/MMP9/MMP2 axis [207]; of the *Celastrus orbiculatus* extract (COE), a mixture of 26 compounds isolated from the Chinese herb *Celastrus orbiculatus* vine, which has shown to have anti-cancer activity and inhibit tumour growth and VM downregulating EphA2 [28]; of niclosamide, which derives from salicylic acid, and has been used worldwide as anti-helminthic drug (for approximately 50 years) and inhibit VM through downregulation of the expression of VEGFA, MMP2, ROCK1 and Cdc42 [208]; and of triptonide, used in a wide variety of inflammatory and autoimmune disorders, isolated from the herb *Tripterygium wilfordii* Hook F, which inhibit VM-related gene expression (VE-cadherin and CXCL2) [209]. It is recognised that the naturally derived compounds are characterised by a wide spectrum of pharmacological activity; therefore, they could be exploited to target the multiple and pleiotropic mechanisms of activation of VM.

## Conclusion

In this review, we focused our efforts on the description of the tight connection between TME and the emergence of VM, which is a clear sign of malignancy, and thus could be used as a prognostic factor in clinical assessments. The overall picture suggests considering VM as the tip of an iceberg. The expression of specific biomarkers helps to identify the presence of VM in a tumour lesion and thus understanding how severe the prognosis is, but the emergence of these signs, above sea level, depends on the extremely complicated network of signalling pathway interactions that take place under sea level. Thus, the huge future challenge would be not only to characterise the distinct pathways driving VM—which may vary from cancer to cancer, and from patient to patient—but also the opportunity to plan a therapeutic strategy that answers to the continuous dynamic adaptation of cancer cells to the effect of the used drugs.

## References

[1] Paget S (1889). The distribution of secondary growths in cancer of the breast. The Lancet.

[2] Albini A, Sporn MB (2007). The tumour microenvironment as a target for chemoprevention. Nature Reviews Cancer.

[3] Hanahan D, Weinberg RA (2011). Hallmarks of cancer: The next generation. Cell.

[4] Andreucci E, Peppicelli S, Carta F, Brisotto G, Biscontin E, Ruzzolini J, Calorini L (2017). Carbonic anhydrase IX inhibition affects viability of cancer cells adapted to extracellular acidosis. Journal of Molecular Medicine (Berlin, Germany).

[5] Vaupel P (2004). Tumor microenvironmental physiology and its implications for radiation oncology. Seminars in Radiation Oncology.

[6] Hashim AI, Zhang X, Wojtkowiak JW, Martinez GV, Gillies RJ (2011). Imaging pH and metastasis. NMR in Biomedicine.

[7] Morita T (1995). Low pH leads to sister-chromatid exchanges and chromosomal aberrations, and its clastogenicity is S-dependent. Mutation Research/Environmental Mutagenesis and Related Subjects.

[8] Tang M, Bolderson E, O’Byrne KJ, Richard DJ (2021). Tumor hypoxia drives genomic instability. Frontiers in Cell and Developmental Biology.

[9] Erler JT, Cawthorne CJ, Williams KJ, Koritzinsky M, Wouters BG, Wilson C, Dive C (2004). Hypoxia-mediated down-regulation of Bid and Bax in tumors occurs via hypoxia-inducible factor 1-dependent and -independent mechanisms and contributes to drug resistance. Molecular and Cellular Biology.

[10] Peppicelli S, Bianchini F, Torre E, Calorini L (2014). Contribution of acidic melanoma cells undergoing epithelial-to-mesenchymal transition to aggressiveness of non-acidic melanoma cells. Clinical & Experimental Metastasis.

[11] Hill RP, Marie-Egyptienne DT, Hedley DW (2009). Cancer stem cells, hypoxia and metastasis. Seminars in Radiation Oncology.

[12] Boedtkjer E, Pedersen SF (2020). The acidic tumor microenvironment as a driver of cancer. Annual Review of Physiology.

[13] Mohyeldin A, Garzón-Muvdi T, Quiñones-Hinojosa A (2010). Oxygen in stem cell biology: A critical component of the stem cell niche. Cell Stem Cell.

[14] Andreucci E, Peppicelli S, Ruzzolini J, Bianchini F, Biagioni A, Papucci L, Calorini L (2020). The acidic tumor microenvironment drives a stem-like phenotype in melanoma cells. Journal of Molecular Medicine.

[15] Zhang, T., Suo, C., Zheng, C., & Zhang, H. (2019). Hypoxia and metabolism in metastasis. In D. M. Gilkes (Ed.), *Hypoxia and cancer metastasis*, *1136*, 87–95. Cham: Springer International Publishing. 10.1007/978-3-030-12734-3_6 10.1007/978-3-030-12734-3_6 31201718

[16] LaMonte G, Tang X, Chen JL-Y, Wu J, Ding C-KC, Keenan MM, Chi J-T (2013). Acidosis induces reprogramming of cellular metabolism to mitigate oxidative stress. Cancer & Metabolism.

[17] Hernández de la Cruz ON, López-González JS, García-Vázquez R, Salinas-Vera YM, Muñiz-Lino MA, Aguilar-Cazares D, Carlos-Reyes Á (2019). Regulation networks driving vasculogenic mimicry in solid tumors. Frontiers in Oncology.

[18] Krishna Priya S, Nagare RP, Sneha VS, Sidhanth C, Bindhya S, Manasa P, Ganesan TS (2016). Tumour angiogenesis–origin of blood vessels: Tumour angiogenesis. International Journal of Cancer.

[19] Peri S, Biagioni A, Versienti G, Andreucci E, Staderini F, Barbato G, Magnelli L (2021). Enhanced vasculogenic capacity induced by 5-fluorouracil chemoresistance in a gastric cancer cell line. International Journal of Molecular Sciences.

[20] Maniotis AJ, Folberg R, Hess A, Seftor EA, Gardner LMG, Pe’er J, Hendrix MJC (1999). Vascular channel formation by human melanoma cells in vivo and in vitro: Vasculogenic mimicry. The American Journal of Pathology.

[21] Maniotis AJ, Folberg R, Hess A, Seftor EA, Gardner LM, Pe’er J, Hendrix MJC (1999). Vascular channel formation by human melanoma cells in vivo and in vitro: Vasculogenic mimicry. The American Journal of Pathology.

[22] Schnegg CI, Yang MH, Ghosh SK, Hsu M-Y (2015). Induction of vasculogenic mimicry overrides VEGF-A silencing and enriches stem-like cancer cells in melanoma. Cancer Research.

[23] He W, Yang G, Liu S, Maghsoudloo M, Shasaltaneh MD, Kaboli PJ, Wen Q (2021). Comparative mRNA/micro-RNA co-expression network drives melanomagenesis by promoting epithelial–mesenchymal transition and vasculogenic mimicry signaling. Translational Oncology.

[24] Mei X, Chen Y-S, Zhang Q-P, Chen F-R, Xi S-Y, Long Y-K, Chen Z-P (2020). Association between glioblastoma cell-derived vessels and poor prognosis of the patients. Cancer Communications (London, England).

[25] Pagano C, Navarra G, Pastorino O, Avilia G, Coppola L, Della Monica R, Laezza C (2021). N6-Isopentenyladenosine hinders the vasculogenic mimicry in human glioblastoma cells through Src-120 catenin pathway modulation and RhoA activity inhibition. International Journal of Molecular Sciences.

[26] Ren K, Yao N, Wang G, Tian L, Ma J, Shi X, Sun X (2014). Vasculogenic mimicry: A new prognostic sign of human osteosarcoma. Human Pathology.

[27] Ren K, Ni Y, Li X, Wang C, Chang Q, Li Y, Zhou J (2019). Expression profiling of long noncoding RNAs associated with vasculogenic mimicry in osteosarcoma. Journal of Cellular Biochemistry.

[28] Chu Z, Shi X, Chen G, He X, Qian Y, Wang H, Chen J (2021). COE inhibits vasculogenic mimicry by targeting EphA2 in hepatocellular carcinoma, a research based on proteomics analysis. Frontiers in Pharmacology.

[29] Li X, Sun B, Zhao X, An J, Zhang Y, Gu Q, Liu F (2020). Function of BMP4 in the formation of vasculogenic mimicry in hepatocellular carcinoma. Journal of Cancer.

[30] Andonegui-Elguera MA, Alfaro-Mora Y, Cáceres-Gutiérrez R, Caro-Sánchez CHS, Herrera LA, Díaz-Chávez J (2020). An overview of vasculogenic mimicry in breast cancer. Frontiers in Oncology.

[31] Shirakawa K, Wakasugi H, Heike Y, Watanabe I, Yamada S, Saito K, Konishi F (2002). Vasculogenic mimicry and pseudo-comedo formation in breast cancer. International Journal of Cancer.

[32] Xia Y, Cai X-Y, Fan J-Q, Zhang L-L, Ren J-H, Li Z-Y, Wu G (2019). The role of sema4D in vasculogenic mimicry formation in non-small cell lung cancer and the underlying mechanisms: Role of sema4D in vasculogenic mimicry formation. International Journal of Cancer.

[33] Niu K, Chen X-W, Qin Y, Zhang L-P, Liao R-X, Sun J-G (2021). Celecoxib blocks vasculogenic mimicry via an off-target effect to radiosensitize lung cancer cells: An experimental study. Frontiers in Oncology.

[34] Kim HS, Won YJ, Shim JH, Kim HJ, Kim J, Hong HN, Kim BS (2019). Morphological characteristics of vasculogenic mimicry and its correlation with EphA2 expression in gastric adenocarcinoma. Scientific Reports.

[35] Song X, An Y, Chen D, Zhang W, Wu X, Li C, Cao H (2021). Microbial metabolite deoxycholic acid promotes vasculogenic mimicry formation in intestinal carcinogenesis. Cancer Science.

[36] Baeten CIM, Hillen F, Pauwels P, de Bruine AP, Baeten CGMI (2009). Prognostic role of vasculogenic mimicry in colorectal cancer. Diseases of the Colon & Rectum.

[37] Liu R, Yang K, Meng C, Zhang Z, Xu Y (2012). Vasculogenic mimicry is a marker of poor prognosis in prostate cancer. Cancer Biology & Therapy.

[38] Wang H, Lin H, Pan J, Mo C, Zhang F, Huang B, Qiu S (2016). Vasculogenic mimicry in prostate cancer: The roles of EphA2 and PI3K. Journal of Cancer.

[39] Luo Q, Wang J, Zhao W, Peng Z, Liu X, Li B, Duan C (2020). Vasculogenic mimicry in carcinogenesis and clinical applications. Journal of Hematology & Oncology.

[40] Yang JP, Liao YD, Mai DM, Xie P, Qiang YY, Zheng LS, Qian CN (2016). Tumor vasculogenic mimicry predicts poor prognosis in cancer patients: A meta-analysis. Angiogenesis.

[41] Zhang X, Zhang J, Zhou H, Fan G, Li Q (2019). Molecular mechanisms and anticancer therapeutic strategies in vasculogenic mimicry. Journal of Cancer.

[42] Delgado-Bellido D, Serrano-Saenz S, Fernández-Cortés M, Oliver FJ (2017). Vasculogenic mimicry signaling revisited: Focus on non-vascular VE-cadherin. Molecular Cancer.

[43] Cao, Z., Bao, M., Miele, L., Sarkar, F. H., Wang, Z., & Zhou, Q. (2013). Tumour vasculogenic mimicry is associated with poor prognosis of human cancer patients: a systemic review and meta-analysis. *European Journal of Cancer (Oxford, England: 1990)*, *49*(18), 3914–3923. 10.1016/j.ejca.2013.07.148 10.1016/j.ejca.2013.07.148 23992642

[44] Shirakawa K, Kobayashi H, Heike Y, Kawamoto S, Brechbiel MW, Kasumi F, Wakasugi H (2002). Hemodynamics in vasculogenic mimicry and angiogenesis of inflammatory breast cancer xenograft. Cancer Research.

[45] Ding J, Jia X, Zuo B, He J, Yang J, He Y (2018). A novel monoclonal antibody targeting a novel epitope of VE-cadherin inhibits vasculogenic mimicry of lung cancer cells. Oncology Reports.

[46] Liu W, Xu G, Jia W, Li J, Ma J, Chen K, Wang X (2011). Prognostic significance and mechanisms of patterned matrix vasculogenic mimicry in hepatocellular carcinoma. Medical Oncology.

[47] Tang H-S, Feng Y-J, Yao L-Q (2009). Angiogenesis, vasculogenesis, and vasculogenic mimicry in ovarian cancer. International Journal of Gynecological Cancer.

[48] Guo Q, Yuan Y, Jin Z, Xu T, Gao Y, Wei H, Hua B (2016). Association between tumor vasculogenic mimicry and the poor prognosis of gastric cancer in China: An updated systematic review and meta-analysis. BioMed Research International.

[49] Fujimoto A, Onodera H, Mori A, Nagayama S, Yonenaga Y, Tachibana T (2006). Tumour plasticity and extravascular circulation in ECV304 human bladder carcinoma cells. Anticancer Research.

[50] Liu Q, Qiao L, Liang N, Xie J, Zhang J, Deng G, Zhang J (2016). The relationship between vasculogenic mimicry and epithelial-mesenchymal transitions. Journal of Cellular and Molecular Medicine.

[51] Andreucci E, Margheri F, Peppicelli S, Bianchini F, Ruzzolini J, Laurenzana A, Calorini L (2021). Glycolysis-derived acidic microenvironment as a driver of endothelial dysfunction in systemic sclerosis. Rheumatology.

[52] Angara K, Borin TF, Arbab AS (2017). Vascular mimicry: A novel neovascularization mechanism driving anti-angiogenic therapy (AAT) resistance in glioblastoma. Translational Oncology.

[53] Serova, M., Tijeras-Raballand, A., Santos, C. D., Martinet, M., Neuzillet, C., Lopez, A., de Gramont, A. (2016). Everolimus affects vasculogenic mimicry in renal carcinoma resistant to sunitinib. *Oncotarget*, *7*(25), 38467–38486. 10.18632/oncotarget.9542 10.18632/oncotarget.9542 PMC5122404 27509260

[54] Sun H, Zhang D, Yao Z, Lin X, Liu J, Gu Q, Sun S (2017). Anti-angiogenic treatment promotes triple-negative breast cancer invasion via vasculogenic mimicry. Cancer Biology & Therapy.

[55] Vasudev NS, Reynolds AR (2014). Anti-angiogenic therapy for cancer: Current progress, unresolved questions and future directions. Angiogenesis.

[56] Lu X-S, Sun W, Ge C-Y, Zhang W-Z, Fan Y-Z (2013). Contribution of the PI3K/MMPs/Ln-5γ2 and EphA2/FAK/Paxillin signaling pathways to tumor growth and vasculogenic mimicry of gallbladder carcinomas. International Journal of Oncology.

[57] Pinto M, Sotomayor P, Carrasco-Avino G, Corvalan A, Owen G (2016). Escaping antiangiogenic therapy: Strategies employed by cancer cells. International Journal of Molecular Sciences.

[58] Gillies RJ, Gatenby RA (2007). Adaptive landscapes and emergent phenotypes: Why do cancers have high glycolysis?. Journal of Bioenergetics and Biomembranes.

[59] Gatenby RA, Gillies RJ (2008). A microenvironmental model of carcinogenesis. Nature Reviews Cancer.

[60] Lugano R, Ramachandran M, Dimberg A (2020). Tumor angiogenesis: Causes, consequences, challenges and opportunities. Cellular and Molecular Life Sciences.

[61] Muz, B., de la Puente, P., Azab, F., & Azab, A. K. (2015). The role of hypoxia in cancer progression, angiogenesis, metastasis, and resistance to therapy. *Hypoxia (Auckland, N.Z.)*, *3*, 83–92. 10.2147/HP.S93413 10.2147/HP.S93413 PMC5045092 27774485

[62] Rajabi M, Mousa S (2017). The role of angiogenesis in cancer treatment. Biomedicines.

[63] Krock BL, Skuli N, Simon MC (2011). Hypoxia-induced angiogenesis: Good and evil. Genes & Cancer.

[64] Hashimoto, T., & Shibasaki, F. (2015). Hypoxia-inducible factor as an angiogenic master switch. *Frontiers in Pediatrics*, *3*. 10.3389/fped.2015.00033 10.3389/fped.2015.00033 PMC4408850 25964891

[65] Ji R-C (2014). Hypoxia and lymphangiogenesis in tumor microenvironment and metastasis. Cancer Letters.

[66] Shi Q, Le X, Wang B, Abbruzzese JL, Xiong Q, He Y, Xie K (2001). Regulation of vascular endothelial growth factor expression by acidosis in human cancer cells. Oncogene.

[67] Scott PAE, Gleadle JM, Bicknell R, Harris AL (1998). Role of the hypoxia sensing system, acidity and reproductive hormones in the variability of vascular endothelial growth factor induction in human breast carcinoma cell lines. International Journal of Cancer.

[68] Peppicelli S, Bianchini F, Contena C, Tombaccini D, Calorini L (2013). Acidic pH via NF-κB favours VEGF-C expression in human melanoma cells. Clinical & Experimental Metastasis.

[69] Skobe M, Hamberg LM, Hawighorst T, Schirner M, Wolf GL, Alitalo K, Detmar M (2001). Concurrent induction of lymphangiogenesis, angiogenesis, and macrophage recruitment by vascular endothelial growth factor-C in melanoma. The American Journal of Pathology.

[70] Nakanishi M, Morita Y, Hata K, Muragaki Y (2016). Acidic microenvironments induce lymphangiogenesis and IL-8 production via TRPV1 activation in human lymphatic endothelial cells. Experimental Cell Research.

[71] Faes, S., Uldry, E., Planche, A., Santoro, T., Pythoud, C., Demartines, N., & Dormond, O. (2016). Acidic pH reduces VEGF-mediated endothelial cell responses by downregulation of VEGFR-2; relevance for anti-angiogenic therapies. *Oncotarget*, *7*(52), 86026–86038. 10.18632/oncotarget.13323 10.18632/oncotarget.13323 PMC5349894 27852069

[72] Mena HA, Lokajczyk A, Dizier B, Strier SE, Voto LS, Boisson-Vidal C, Negrotto S (2014). Acidic preconditioning improves the proangiogenic responses of endothelial colony forming cells. Angiogenesis.

[73] Mena HA, Zubiry PR, Dizier B, Schattner M, Boisson-Vidal C, Negrotto S (2018). Acidic preconditioning of endothelial colony-forming cells (ECFC) promote vasculogenesis under proinflammatory and high glucose conditions in vitro and in vivo. Stem Cell Research & Therapy.

[74] Tomaskovic-Crook E, Thompson EW, Thiery JP (2009). Epithelial to mesenchymal transition and breast cancer. Breast Cancer Research.

[75] Polyak K, Weinberg RA (2009). Transitions between epithelial and mesenchymal states: Acquisition of malignant and stem cell traits. Nature Reviews Cancer.

[76] Rouschop KMA, van den Beucken T, Dubois L, Niessen H, Bussink J, Savelkouls K, Wouters BG (2010). The unfolded protein response protects human tumor cells during hypoxia through regulation of the autophagy genes MAP1LC3B and ATG5. Journal of Clinical Investigation.

[77] Wojtkowiak JW, Rothberg JM, Kumar V, Schramm KJ, Haller E, Proemsey JB, Gillies RJ (2012). Chronic autophagy is a cellular adaptation to tumor acidic pH microenvironments. Cancer Research.

[78] Rohwer N, Welzel M, Daskalow K, Pfander D, Wiedenmann B, Detjen K, Cramer T (2008). Hypoxia-inducible factor 1α mediates anoikis resistance via suppression of α5 integrin. Cancer Research.

[79] Peppicelli S, Ruzzolini J, Bianchini F, Andreucci E, Nediani C, Laurenzana A, Calorini L (2019). Anoikis resistance as a further trait of acidic-adapted melanoma cells. Journal of Oncology.

[80] Emami Nejad A, Najafgholian S, Rostami A, Sistani A, Shojaeifar S, Esparvarinha M, Manian M (2021). The role of hypoxia in the tumor microenvironment and development of cancer stem cell: A novel approach to developing treatment. Cancer Cell International.

[81] Mathieu J, Zhang Z, Zhou W, Wang AJ, Heddleston JM, Pinna CMA, Ruohola-Baker H (2011). HIF induces human embryonic stem cell markers in cancer cells. Cancer Research.

[82] Pietras A, Hansford LM, Johnsson AS, Bridges E, Sjölund J, Gisselsson D, Påhlman S (2009). HIF-2α maintains an undifferentiated state in neural crest-like human neuroblastoma tumor-initiating cells. Proceedings of the National Academy of Sciences.

[83] Li Z, Bao S, Wu Q, Wang H, Eyler C, Sathornsumetee S, Rich JN (2009). Hypoxia-inducible factors regulate tumorigenic capacity of glioma stem cells. Cancer Cell.

[84] Huang S, Tang Y, Peng X, Cai X, Wa Q, Ren D, Huang S (2016). Acidic extracellular pH promotes prostate cancer bone metastasis by enhancing PC-3 stem cell characteristics, cell invasiveness and VEGF-induced vasculogenesis of BM-EPCs. Oncology Reports.

[85] Filatova A, Seidel S, Böğürcü N, Gräf S, Garvalov BK, Acker T (2016). Acidosis acts through HSP90 in a PHD/VHL-independent manner to promote HIF function and stem cell maintenance in glioma. Cancer Research.

[86] Hu P, Li S, Tian N, Wu F, Hu Y, Li D, Peng X (2019). Acidosis enhances the self-renewal and mitochondrial respiration of stem cell-like glioma cells through CYP24A1-mediated reduction of vitamin D. Cell Death & Disease.

[87] Avnet S, Di Pompo G, Chano T, Errani C, Ibrahim-Hashim A, Gillies RJ, Baldini N (2017). Cancer-associated mesenchymal stroma fosters the stemness of osteosarcoma cells in response to intratumoral acidosis via NF-κB activation: Tumor acidic microenvironment fosters osteosarcoma stemness via mesenchymal stroma. International Journal of Cancer.

[88] Zakaria N, Mohd Yusoff N, Zakaria Z, Widera D, Yahaya BH (2018). Inhibition of NF-κB signaling reduces the stemness characteristics of lung cancer stem cells. Frontiers in Oncology.

[89] Gong C, Bauvy C, Tonelli G, Yue W, Deloménie C, Nicolas V, Mehrpour M (2013). Beclin 1 and autophagy are required for the tumorigenicity of breast cancer stem-like/progenitor cells. Oncogene.

[90] Peppicelli S, Bianchini F, Calorini L (2014). Extracellular acidity, a “reappreciated” trait of tumor environment driving malignancy: Perspectives in diagnosis and therapy. Cancer and Metastasis Reviews.

[91] Harris AL (2002). Hypoxia — a key regulatory factor in tumour growth. Nature Reviews Cancer.

[92] Codony VL, Tavassoli M (2021). Hypoxia-induced therapy resistance: Available hypoxia-targeting strategies and current advances in head and neck cancer. Translational Oncology.

[93] Thews O, Gassner B, Kelleher DK, Schwerd G, Gekle M (2006). Impact of extracellular acidity on the activity of P-glycoprotein and the cytotoxicity of chemotherapeutic drugs. Neoplasia.

[94] Mahoney BP, Raghunand N, Baggett B, Gillies RJ (2003). Tumor acidity, ion trapping and chemotherapeutics. Biochemical Pharmacology.

[95] Gerweck LE, Vijayappa S, Kozin S (2006). Tumor pH controls the *in vivo* efficacy of weak acid and base chemotherapeutics. Molecular Cancer Therapeutics.

[96] Raghunand N, He X, van Sluis R, Mahoney B, Baggett B, Taylor CW, Gillies RJ (1999). Enhancement of chemotherapy by manipulation of tumour pH. British Journal of Cancer.

[97] Rausch V, Liu L, Apel A, Rettig T, Gladkich J, Labsch S, Herr I (2012). Autophagy mediates survival of pancreatic tumour-initiating cells in a hypoxic microenvironment: Autophagy in pancreatic tumourigenic cells. The Journal of Pathology.

[98] Amaravadi RK, Yu D, Lum JJ, Bui T, Christophorou MA, Evan GI, Thompson CB (2007). Autophagy inhibition enhances therapy-induced apoptosis in a Myc-induced model of lymphoma. Journal of Clinical Investigation.

[99] Lomonaco SL, Finniss S, Xiang C, DeCarvalho A, Umansky F, Kalkanis SN, Brodie C (2009). The induction of autophagy by γ-radiation contributes to the radioresistance of glioma stem cells. International Journal of Cancer.

[100] Daskalaki I, Gkikas I, Tavernarakis N (2018). Hypoxia and selective autophagy in cancer development and therapy. Frontiers in Cell and Developmental Biology.

[101] Bellot G, Garcia-Medina R, Gounon P, Chiche J, Roux D, Pouysségur J, Mazure NM (2009). Hypoxia-induced autophagy is mediated through hypoxia-inducible factor induction of BNIP3 and BNIP3L via their BH3 domains. Molecular and Cellular Biology.

[102] Marino ML, Fais S, Djavaheri-Mergny M, Villa A, Meschini S, Lozupone F, De Milito A (2010). Proton pump inhibition induces autophagy as a survival mechanism following oxidative stress in human melanoma cells. Cell Death & Disease.

[103] Wojtkowiak JW, Gillies RJ (2012). Autophagy on acid. Autophagy.

[104] Abou Khouzam R, Goutham HV, Zaarour RF, Chamseddine AN, Francis A, Buart S, Chouaib S (2020). Integrating tumor hypoxic stress in novel and more adaptable strategies for cancer immunotherapy. Seminars in Cancer Biology.

[105] Wang JX, Choi SYC, Niu X, Kang N, Xue H, Killam J, Wang Y (2020). Lactic acid and an acidic tumor microenvironment suppress anticancer immunity. International Journal of Molecular Sciences.

[106] Damgaci S, Ibrahim-Hashim A, Enriquez-Navas PM, Pilon-Thomas S, Guvenis A, Gillies RJ (2018). Hypoxia and acidosis: Immune suppressors and therapeutic targets. Immunology.

[107] Huber V, Camisaschi C, Berzi A, Ferro S, Lugini L, Triulzi T, Rivoltini L (2017). Cancer acidity: An ultimate frontier of tumor immune escape and a novel target of immunomodulation. Seminars in Cancer Biology.

[108] Noman, M. Z., Hasmim, M., Messai, Y., Terry, S., Kieda, C., Janji, B., & Chouaib, S. (2015). Hypoxia: a key player in antitumor immune response. A review in the theme: cellular responses to hypoxia. *American Journal of Physiology-Cell Physiology*, *309*(9), C569–C579. 10.1152/ajpcell.00207.2015 10.1152/ajpcell.00207.2015 PMC4628936 26310815

[109] Feichtinger RG, Lang R (2019). Targeting L-lactate metabolism to overcome resistance to immune therapy of melanoma and other tumor entities. Journal of Oncology.

[110] Hu M, Li Y, Lu Y, Wang M, Li Y, Wang C, Zhao H (2021). The regulation of immune checkpoints by the hypoxic tumor microenvironment. Peer J.

[111] Hirschhaeuser F, Sattler UGA, Mueller-Klieser W (2011). Lactate: A metabolic key player in cancer. Cancer Research.

[112] Calcinotto A, Filipazzi P, Grioni M, Iero M, De Milito A, Ricupito A, Rivoltini L (2012). Modulation of microenvironment acidity reverses anergy in human and murine tumor-infiltrating T lymphocytes. Cancer Research.

[113] Sun J, Zhang Y, Yang M, Zhang Y, Xie Q, Li Z, Qu X (2010). Hypoxia induces T-cell apoptosis by inhibiting chemokine C receptor 7 expression: The role of adenosine receptor A2. Cellular & Molecular Immunology.

[114] Atkuri KR, Herzenberg LA, Niemi A-K, Cowan T, Herzenberg LA (2007). Importance of culturing primary lymphocytes at physiological oxygen levels. Proceedings of the National Academy of Sciences.

[115] Lukashev, D., Klebanov, B., Kojima, H., Grinberg, A., Ohta, A., Berenfeld, L., Sitkovsky, M. (2006). Cutting edge: hypoxia-inducible factor 1α and its activation-inducible short isoform I.1 negatively regulate functions of CD4^+^ and CD8^+^ T lymphocytes. *The Journal of Immunology*, *177*(8), 4962–4965. 10.4049/jimmunol.177.8.4962 10.4049/jimmunol.177.8.4962 17015677

[116] Flück K, Breves G, Fandrey J, Winning S (2016). Hypoxia-inducible factor 1 in dendritic cells is crucial for the activation of protective regulatory T cells in murine colitis. Mucosal Immunology.

[117] Gottfried E, Kunz-Schughart LA, Ebner S, Mueller-Klieser W, Hoves S, Andreesen R, Kreutz M (2006). Tumor-derived lactic acid modulates dendritic cell activation and antigen expression. Blood.

[118] Mancino A, Schioppa T, Larghi P, Pasqualini F, Nebuloni M, Chen I-H, Sica A (2008). Divergent effects of hypoxia on dendritic cell functions. Blood.

[119] Severin T, Müller B, Giese G, Uhl B, Wolf B, Hauschildt S, Kreutz W (1994). pH-Dependent LAK cell cytotoxicity. Tumor Biology.

[120] Ishizaka S, Kimoto M, Tsujii T (1992). Defect in generation of LAK cell activity under oxygen-limited conditions. Immunology Letters.

[121] Liao Y-P (2007). Modification of the tumor microenvironment to enhance immunity. Frontiers in Bioscience.

[122] Solocinski K, Padget MR, Fabian KP, Wolfson B, Cecchi F, Hembrough T, Hodge JW (2020). Overcoming hypoxia-induced functional suppression of NK cells. Journal for ImmunoTherapy of Cancer.

[123] Colegio OR, Chu N-Q, Szabo AL, Chu T, Rhebergen AM, Jairam V, Medzhitov R (2014). Functional polarization of tumour-associated macrophages by tumour-derived lactic acid. Nature.

[124] Ke X, Chen C, Song Y, Cai Q, Li J, Tang Y, Liu D (2019). Hypoxia modifies the polarization of macrophages and their inflammatory microenvironment, and inhibits malignant behavior in cancer cells. Oncology Letters.

[125] Crowther M, Brown NJ, Bishop ET, Lewis CE (2001). Microenvironmental influence on macrophage regulation of angiogenesis in wounds and malignant tumors. Journal of Leukocyte Biology.

[126] Husain Z, Huang Y, Seth P, Sukhatme VP (2013). Tumor-derived lactate modifies antitumor immune response: Effect on myeloid-derived suppressor cells and NK cells. The Journal of Immunology.

[127] Pilon-Thomas S, Kodumudi KN, El-Kenawi AE, Russell S, Weber AM, Luddy K, Gillies RJ (2016). Neutralization of tumor acidity improves antitumor responses to immunotherapy. Cancer Research.

[128] Vishvakarma NK, Singh SM (2010). Immunopotentiating effect of proton pump inhibitor pantoprazole in a lymphoma-bearing murine host: Implication in antitumor activation of tumor-associated macrophages. Immunology Letters.

[129] Bergers G, Fendt S-M (2021). The metabolism of cancer cells during metastasis. Nature Reviews Cancer.

[130] Kim J, Tchernyshyov I, Semenza GL, Dang CV (2006). HIF-1-mediated expression of pyruvate dehydrogenase kinase: A metabolic switch required for cellular adaptation to hypoxia. Cell Metabolism.

[131] Semenza GL, Jiang B-H, Leung SW, Passantino R, Concordet J-P, Maire P, Giallongo A (1996). Hypoxia response elements in the aldolase A, enolase 1, and lactate dehydrogenase A gene promoters contain essential binding sites for hypoxia-inducible factor 1. Journal of Biological Chemistry.

[132] Trojan, S. E., Piwowar, M., Ostrowska, B., Laidler, P., & Kocemba-Pilarczyk, K. A. (2018). Analysis of malignant melanoma cell lines exposed to hypoxia reveals the importance of *PFKFB4* overexpression for disease progression. *Anticancer Research*, *38*(12), 6745–6752. 10.21873/anticanres.13044 10.21873/anticanres.13044 30504385

[133] Schito L, Semenza GL (2016). Hypoxia-inducible factors: Master regulators of cancer progression. Trends in Cancer.

[134] Sun RC, Denko NC (2014). Hypoxic regulation of glutamine metabolism through HIF1 and SIAH2 supports lipid synthesis that is necessary for tumor growth. Cell Metabolism.

[135] Peppicelli, S., Toti, A., Giannoni, E., Bianchini, F., Margheri, F., Del Rosso, M., & Calorini, L. (2016). Metformin is also effective on lactic acidosis-exposed melanoma cells switched to oxidative phosphorylation. *Cell Cycle (Georgetown, Tex.)*, *15*(14), 1908–1918. 10.1080/15384101.2016.1191706 10.1080/15384101.2016.1191706 PMC4968910 27266957

[136] Andreucci E, Pietrobono S, Peppicelli S, Ruzzolini J, Bianchini F, Biagioni A, Calorini L (2018). SOX2 as a novel contributor of oxidative metabolism in melanoma cells. Cell Communication and Signaling: CCS.

[137] Mazzio EA, Boukli N, Rivera N, Soliman KFA (2012). Pericellular pH homeostasis is a primary function of the Warburg effect: Inversion of metabolic systems to control lactate steady state in tumor cells. Cancer Science.

[138] Corbet C, Pinto A, Martherus R, Santiago de Jesus JP, Polet F, Feron O (2016). Acidosis drives the reprogramming of fatty acid metabolism in cancer cells through changes in mitochondrial and histone acetylation. Cell Metabolism.

[139] Corbet C, Draoui N, Polet F, Pinto A, Drozak X, Riant O, Feron O (2014). The SIRT1/HIF2α Axis drives reductive glutamine metabolism under chronic acidosis and alters tumor response to therapy. Cancer Research.

[140] Tang X, Lucas JE, Chen JL-Y, LaMonte G, Wu J, Wang MC, Chi J-T (2012). Functional interaction between responses to lactic acidosis and hypoxia regulates genomic transcriptional outputs. Cancer Research.

[141] Giannotta M, Trani M, Dejana E (2013). VE-cadherin and endothelial adherens junctions: Active guardians of vascular integrity. Developmental Cell.

[142] Pasquale EB (2010). Eph receptors and ephrins in cancer: Bidirectional signalling and beyond. Nature Reviews. Cancer.

[143] Seftor REB, Hess AR, Seftor EA, Kirschmann DA, Hardy KM, Margaryan NV, Hendrix MJC (2012). Tumor cell vasculogenic mimicry. The American Journal of Pathology.

[144] Hess AR, Postovit L-M, Margaryan NV, Seftor EA, Schneider GB, Seftor REB, Hendrix MJC (2005). Focal adhesion kinase promotes the aggressive melanoma phenotype. Cancer Research.

[145] Andreucci E, Laurenzana A, Peppicelli S, Biagioni A, Margheri F, Ruzzolini J, Calorini L (2022). uPAR Controls vasculogenic mimicry ability expressed by drug-resistant melanoma cells. Oncology Research.

[146] Cao W, Xu C, Li X, Yang X (2019). Twist1 promotes astrocytoma development by stimulating vasculogenic mimicry. Oncology Letters.

[147] Sun J, Sun B, Sun R, Zhu D, Zhao X, Zhang Y, Zhang D (2017). HMGA2 promotes vasculogenic mimicry and tumor aggressiveness by upregulating Twist1 in gastric carcinoma. Scientific Reports.

[148] Liu K, Sun B, Zhao X, Wang X, Li Y, Qiu Z, Zhao N (2015). Hypoxia promotes vasculogenic mimicry formation by the Twist1-Bmi1 connection in hepatocellular carcinoma. International Journal of Molecular Medicine.

[149] Frank NY, Schatton T, Kim S, Zhan Q, Wilson BJ, Ma J, Frank MH (2011). VEGFR-1 Expressed by malignant melanoma-initiating cells is required for tumor growth. Cancer Research.

[150] Ayala-Domínguez L, Olmedo-Nieva L, Muñoz-Bello JO, Contreras-Paredes A, Manzo-Merino J, Martínez-Ramírez I, Lizano M (2019). Mechanisms of vasculogenic mimicry in ovarian cancer. Frontiers in Oncology.

[151] Wang J-Y, Sun T, Zhao X-L, Zhang S-W, Zhang D-F, Gu Q, Sun B-C (2008). Functional significance of VEGF-a in human ovarian carcinoma: Role in vasculogenic mimicry. Cancer Biology & Therapy.

[152] Basu GD, Liang WS, Stephan DA, Wegener LT, Conley CR, Pockaj BA, Mukherjee P (2006). A novel role for cyclooxygenase-2 in regulating vascular channel formation by human breast cancer cells. Breast Cancer Research: BCR.

[153] Basu GD, Pathangey LB, Tinder TL, Gendler SJ, Mukherjee P (2005). Mechanisms underlying the growth inhibitory effects of the cyclo-oxygenase-2 inhibitor celecoxib in human breast cancer cells. Breast Cancer Research: BCR.

[154] Pai R, Soreghan B, Szabo IL, Pavelka M, Baatar D, Tarnawski AS (2002). Prostaglandin E2 transactivates EGF receptor: A novel mechanism for promoting colon cancer growth and gastrointestinal hypertrophy. Nature Medicine.

[155] Liu X, Lv Z, Zhou S, Kan S, Liu X, Jing P, Xu W (2021). MTDH in macrophages promotes the vasculogenic mimicry via VEGFA-165/Flt-1 signaling pathway in head and neck squamous cell carcinoma. International Immunopharmacology.

[156] Shi L, Lei D, Ma C, Xu F, Li Y, Wang Y, Pan XL (2010). Clinicopathological implications of tumour-associated macrophages and vascularization in sinonasal melanoma. The Journal of International Medical Research.

[157] Spinella F, Caprara V, Di Castro V, Rosanò L, Cianfrocca R, Natali PG, Bagnato A (2013). Endothelin-1 induces the transactivation of vascular endothelial growth factor receptor-3 and modulates cell migration and vasculogenic mimicry in melanoma cells. Journal of Molecular Medicine (Berlin, Germany).

[158] Petersen LC, Sprecher CA, Foster DC, Blumberg H, Hamamoto T, Kisiel W (1996). Inhibitory properties of a novel human Kunitz-type protease inhibitor homologous to tissue factor pathway inhibitor. Biochemistry.

[159] Miyagi Y, Koshikawa N, Yasumitsu H, Miyagi E, Hirahara F, Aoki I, Miyazaki K (1994). cDNA cloning and mRNA expression of a serine proteinase inhibitor secreted by cancer cells: Identification as placental protein 5 and tissue factor pathway inhibitor-2. Journal of Biochemistry.

[160] Rao CN, Reddy P, Liu Y, O’Toole E, Reeder D, Foster DC, Woodley DT (1996). Extracellular matrix-associated serine protease inhibitors (Mr 33,000, 31,000, and 27,000) are single-gene products with differential glycosylation: CDNA cloning of the 33-kDa inhibitor reveals its identity to tissue factor pathway inhibitor-2. Archives of Biochemistry and Biophysics.

[161] Ruf W, Seftor EA, Petrovan RJ, Weiss RM, Gruman LM, Margaryan NV, Hendrix MJC (2003). Differential role of tissue factor pathway inhibitors 1 and 2 in melanoma vasculogenic mimicry. Cancer Research.

[162] Zang M, Hu L, Zhang B, Zhu Z, Li J, Zhu Z, Liu B (2017). Luteolin suppresses angiogenesis and vasculogenic mimicry formation through inhibiting Notch1-VEGF signaling in gastric cancer. Biochemical and Biophysical Research Communications.

[163] Vartanian A, Gatsina G, Grigorieva I, Solomko E, Dombrovsky V, Baryshnikov A, Stepanova E (2013). The involvement of Notch signaling in melanoma vasculogenic mimicry. Clinical and Experimental Medicine.

[164] Cheng, R., Cai, X.-R., Ke, K., & Chen, Y.-L. (2017). Notch4 inhibition suppresses invasion and vasculogenic mimicry formation of hepatocellular carcinoma cells. *Journal of Huazhong University of Science and Technology. Medical Sciences = Hua Zhong Ke Ji Da Xue Xue Bao. Yi Xue Ying De Wen Ban = Huazhong Keji Daxue Xuebao. Yixue Yingdewen Ban*, *37*(5), 719–725. 10.1007/s11596-017-1794-9 10.1007/s11596-017-1794-9 29058285

[165] Hardy KM, Kirschmann DA, Seftor EA, Margaryan NV, Postovit L-M, Strizzi L, Hendrix MJC (2010). Regulation of the embryonic morphogen Nodal by Notch4 facilitates manifestation of the aggressive melanoma phenotype. Cancer Research.

[166] Qi L, Song W, Liu Z, Zhao X, Cao W, Sun B (2015). Wnt3a Promotes the vasculogenic mimicry formation of colon cancer via Wnt/β-catenin signaling. International Journal of Molecular Sciences.

[167] Yao N, Ren K, Gu XJ, Wu SJ, Shi X, Chang Q, Zhou J (2020). Identification of potential crucial genes associated with vasculogenic mimicry in human osteosarcoma based on gene expression profile. Neoplasma.

[168] Xie W, Zhao H, Wang F, Wang Y, He Y, Wang T, Huang G (2021). A novel humanized Frizzled-7-targeting antibody enhances antitumor effects of bevacizumab against triple-negative breast cancer via blocking Wnt/β-catenin signaling pathway. Journal of Experimental & Clinical Cancer Research: CR.

[169] Shin S-U, Cho H-M, Das R, Gil-Henn H, Ramakrishnan S, Al Bayati A, Rosenblatt JD (2021). Inhibition of vasculogenic mimicry and angiogenesis by an anti-EGFR IgG1-human endostatin-P125A fusion protein reduces triple negative breast cancer metastases. Cells.

[170] Geng B, Zhu Y, Yuan Y, Bai J, Dou Z, Sui A, Luo W (2021). Artesunate suppresses choroidal melanoma vasculogenic mimicry formation and angiogenesis via the Wnt/CaMKII signaling axis. Frontiers in Oncology.

[171] Huang M, Ke Y, Sun X, Yu L, Yang Z, Zhang Y, Huang S (2014). Mammalian target of rapamycin signaling is involved in the vasculogenic mimicry of glioma via hypoxia-inducible factor-1α. Oncology Reports.

[172] Tang N-N, Zhu H, Zhang H-J, Zhang W-F, Jin H-L, Wang L, Shi R-H (2014). HIF-1α induces VE-cadherin expression and modulates vasculogenic mimicry in esophageal carcinoma cells. World Journal of Gastroenterology.

[173] Comito G, Calvani M, Giannoni E, Bianchini F, Calorini L, Torre E, Chiarugi P (2011). HIF-1α stabilization by mitochondrial ROS promotes Met-dependent invasive growth and vasculogenic mimicry in melanoma cells. Free Radical Biology and Medicine.

[174] Seftor REB, Hess AR, Seftor EA, Kirschmann DA, Hardy KM, Margaryan NV, Hendrix MJC (2012). Tumor cell vasculogenic mimicry: From controversy to therapeutic promise. The American Journal of Pathology.

[175] Zhao N, Sun B, Sun T, Ma Y, Zhao X, Liu Z, Gu Q (2012). Hypoxia-induced vasculogenic mimicry formation via VE-cadherin regulation by Bcl-2. Medical Oncology (Northwood, London, England).

[176] Mao X-G, Xue X-Y, Wang L, Zhang X, Yan M, Tu Y-Y, Song S-J (2013). CDH5 is specifically activated in glioblastoma stemlike cells and contributes to vasculogenic mimicry induced by hypoxia. Neuro-Oncology.

[177] D’Aguanno S, Mallone F, Marenco M, Del Bufalo D, Moramarco A (2021). Hypoxia-dependent drivers of melanoma progression. Journal of Experimental & Clinical Cancer Research: CR.

[178] Li, S., Meng, W., Guan, Z., Guo, Y., & Han, X. (2016). The hypoxia-related signaling pathways of vasculogenic mimicry in tumor treatment. *Biomedicine & Pharmacotherapy = Biomedecine & Pharmacotherapie*, *80*, 127–135. 10.1016/j.biopha.2016.03.010 10.1016/j.biopha.2016.03.010 27133049

[179] Krishnamachary B, Berg-Dixon S, Kelly B, Agani F, Feldser D, Ferreira G, Semenza GL (2003). Regulation of colon carcinoma cell invasion by hypoxia-inducible factor 1. Cancer Research.

[180] Li Y, Sun B, Zhao X, Zhang D, Wang X, Zhu D, Ban X (2015). Subpopulations of uPAR+ contribute to vasculogenic mimicry and metastasis in large cell lung cancer. Experimental and Molecular Pathology.

[181] Bedal KB, Grässel S, Spanier G, Reichert TE, Bauer RJ (2015). The NC11 domain of human collagen XVI induces vasculogenic mimicry in oral squamous cell carcinoma cells. Carcinogenesis.

[182] Hugdahl E, Bachmann IM, Schuster C, Ladstein RG, Akslen LA (2019). Prognostic value of uPAR expression and angiogenesis in primary and metastatic melanoma. PLoS One.

[183] Peppicelli S, Bianchini F, Calorini L (2015). Metabolic reprogramming as a continuous changing behavior of tumor cells. Tumour Biology: The Journal of the International Society for Oncodevelopmental Biology and Medicine.

[184] Ruzzolini J, Peppicelli S, Andreucci E, Bianchini F, Margheri F, Laurenzana A, Calorini L (2017). Everolimus selectively targets vemurafenib resistant BRAFV600E melanoma cells adapted to low pH. Cancer Letters.

[185] Sun B, Zhang S, Zhang D, Du J, Guo H, Zhao X, Hao X (2006). Vasculogenic mimicry is associated with high tumor grade, invasion and metastasis, and short survival in patients with hepatocellular carcinoma. Oncology Reports.

[186] Wang S, Ke Y, Lu G, Song Z, Yu L, Xiao S, Hu C (2013). Vasculogenic mimicry is a prognostic factor for postoperative survival in patients with glioblastoma. Journal of Neuro-Oncology.

[187] Wu S, Yu L, Wang D, Zhou L, Cheng Z, Chai D, Tao Y (2012). Aberrant expression of CD133 in non-small cell lung cancer and its relationship to vasculogenic mimicry. BMC Cancer.

[188] Han D-S, Lee E-O (2022). Sp1 plays a key role in vasculogenic mimicry of human prostate cancer cells. International Journal of Molecular Sciences.

[189] Lezcano, C., Kleffel, S., Lee, N., Larson, A. R., Zhan, Q., DoRosario, A., Murphy, G. F. (2014). Merkel cell carcinoma expresses vasculogenic mimicry: demonstration in patients and experimental manipulation in xenografts. *Laboratory Investigation; a Journal of Technical Methods and Pathology*, *94*(10), 1092–1102. 10.1038/labinvest.2014.99 10.1038/labinvest.2014.99 PMC4236190 25111691

[190] Belotti D, Pinessi D, Taraboletti G (2021). Alternative vascularization mechanisms in tumor resistance to therapy. Cancers.

[191] van der Schaft DWJ, Seftor REB, Seftor EA, Hess AR, Gruman LM, Kirschmann DA, Hendrix MJC (2004). Effects of angiogenesis inhibitors on vascular network formation by human endothelial and melanoma cells. JNCI Journal of the National Cancer Institute.

[192] Shimizu S, Kawahara R, Simizu S (2021). Methionine aminopeptidase-2 is a pivotal regulator of vasculogenic mimicry. Oncology Reports.

[193] He M, Yang H, Shi H, Hu Y, Chang C, Liu S, Yeh S (2022). Sunitinib increases the cancer stem cells and vasculogenic mimicry formation via modulating the lncRNA-ECVSR/ERβ/Hif2-α signaling. Cancer Letters.

[194] Ruffini F, Graziani G, Levati L, Tentori L, D’Atri S, Lacal PM (2015). Cilengitide downmodulates invasiveness and vasculogenic mimicry of neuropilin 1 expressing melanoma cells through the inhibition of αvβ5 integrin: Cilengitide inhibits melanoma invasiveness and vasculogenic mimicry. International Journal of Cancer.

[195] Wang Y, Tong L, Wang J, Luo J, Tang J, Zhong L, Wang Y (2019). cRGD-functionalized nanoparticles for combination therapy of anti-endothelium dependent vessels and anti-vasculogenic mimicry to inhibit the proliferation of ovarian cancer. Acta Biomaterialia.

[196] Camorani S, Crescenzi E, Gramanzini M, Fedele M, Zannetti A, Cerchia L (2017). Aptamer-mediated impairment of EGFR-integrin αvβ3 complex inhibits vasculogenic mimicry and growth of triple-negative breast cancers. Scientific Reports.

[197] Bianchini F, De Santis A, Portioli E, Russo Krauss I, Battistini L, Curti C, Sartori A (2019). Integrin-targeted AmpRGD sunitinib liposomes as integrated antiangiogenic tools. Nanomedicine: Nanotechnology Biology and Medicine.

[198] Bianchini F, Portioli E, Ferlenghi F, Vacondio F, Andreucci E, Biagioni A, Sartori A (2019). Cell-targeted c(AmpRGD)-sunitinib molecular conjugates impair tumor growth of melanoma. Cancer Letters.

[199] Sartori A, Portioli E, Battistini L, Calorini L, Pupi A, Vacondio F, Zanardi F (2017). Synthesis of novel c(AmpRGD)-sunitinib dual conjugates as molecular tools targeting the αvβ3 integrin/VEGFR2 couple and impairing tumor-associated angiogenesis. Journal of Medicinal Chemistry.

[200] Liu, W., Lv, C., Zhang, B., Zhou, Q., & Cao, Z. (2017). MicroRNA-27b functions as a new inhibitor of ovarian cancer-mediated vasculogenic mimicry through suppression of VE-cadherin expression. *RNA (New York, N.Y.)*, *23*(7), 1019–1027. 10.1261/rna.059592.116 10.1261/rna.059592.116 PMC5473136 28396577

[201] Langer EM, Kendsersky ND, Daniel CJ, Kuziel GM, Pelz C, Murphy KM, Sears RC (2018). ZEB1-repressed microRNAs inhibit autocrine signaling that promotes vascular mimicry of breast cancer cells. Oncogene.

[202] Fu R, Du W, Ding Z, Wang Y, Li Y, Zhu J, Huang J (2021). HIF-1α promoted vasculogenic mimicry formation in lung adenocarcinoma through NRP1 upregulation in the hypoxic tumor microenvironment. Cell Death & Disease.

[203] Wang B, Zhang H, Wei L, Li Y (2022). Expression of Dickkopf-1 and Twist2 in cervical squamous cell carcinoma and their correlation with vasculogenic mimicry. Journal of Healthcare Engineering.

[204] Treps L, Faure S, Clere N (2021). Vasculogenic mimicry, a complex and devious process favoring tumorigenesis – interest in making it a therapeutic target. Pharmacology & Therapeutics.

[205] Haiaty S, Rashidi M-R, Akbarzadeh M, Maroufi NF, Yousefi B, Nouri M (2020). Targeting vasculogenic mimicry by phytochemicals: A potential opportunity for cancer therapy. IUBMB Life.

[206] Chiablaem K, Lirdprapamongkol K, Keeratichamroen S, Surarit R, Svasti J (2014). Curcumin suppresses vasculogenic mimicry capacity of hepatocellular carcinoma cells through STAT3 and PI3K/AKT inhibition. Anticancer Research.

[207] Guo J-Q, Zheng Q-H, Chen H, Chen L, Xu J-B, Chen M-Y, Lin S (2014). Ginsenoside Rg3 inhibition of vasculogenic mimicry in pancreatic cancer through downregulation of VE-cadherin/EphA2/MMP9/MMP2 expression. International Journal of Oncology.

[208] Li X, Yang Z, Han Z, Wen Y, Ma Z, Wang Y (2018). Niclosamide acts as a new inhibitor of vasculogenic mimicry in oral cancer through upregulation of miR-124 and downregulation of STAT3. Oncology Reports.

[209] Han H, Du L, Cao Z, Zhang B, Zhou Q (2018). Triptonide potently suppresses pancreatic cancer cell-mediated vasculogenic mimicry by inhibiting expression of VE-cadherin and chemokine ligand 2 genes. European Journal of Pharmacology.

